# The oral [^13^C]bicarbonate technique for measurement of short-term energy expenditure of sled dogs and their physiological response to diets with different fat:carbohydrate ratios

**DOI:** 10.1017/jns.2015.23

**Published:** 2015-10-12

**Authors:** Caroline Larsson, Øystein Ahlstrøm, Peter Junghans, Rasmus B. Jensen, Dominique Blache, Anne-Helene Tauson

**Affiliations:** 1 Department of Veterinary Clinical and Animal Sciences, Faculty of Health and Medical Sciences, University of Copenhagen, Frederiksberg, Denmark; 2 Department of Animal and Aquacultural Sciences, Faculty of Veterinary Medicine and Biosciences, Norwegian University of Life Sciences, Ås, Norway; 3 Institute of Nutritional Physiology ‘Oskar Kellner’, Leibniz Institute for Farm Animal Biology, Dummerstorf, Germany; 4 School of Animal Biology, University of Western Australia, Perth, Australia

**Keywords:** Energy expenditure, ^13^C, Dogs, Diet, Digestibility, ATTD, apparent total tract digestibility, BW, body weight, BW^0·75^, metabolic body weight, EE, energy expenditure, HR, heart rate, IGF-1, insulin-like growth factor 1, ME, metabolisable energy, o^13^CBT, oral [^13^C]bicarbonate technique, PF diet, protein–fat diet, PFC diet, protein–fat-carbohydrate diet, ppm, parts per million, RF, fractional ^13^C recovery in breath CO_2_, RQ, respiratory quotient, *T*_R_, rectal temperature

## Abstract

The oral [^13^C]bicarbonate technique (o^13^CBT) was assessed for the determination of short-term energy expenditure (EE) under field conditions. A total of eight Alaskan huskies were fed two experimental diets in a cross-over experiment including two periods of 3 weeks. Effects of diets on EE, apparent total tract digestibility (ATTD) and on plasma hormones, blood lactate and glucose were furthermore investigated. The percentages of metabolisable energy derived from protein (P), fat (F) and carbohydrates (C) were 26:58:16 in the PFC diet and 24:75:1 in the PF diet. Measurements of EE were performed in the post-absorptive state during rest. Blood samples were collected during rest and exercise and ATTD was determined after days with rest and with exercise. EE was higher (*P* < 0·01) in period 2 than in period 1 (68 *v.* 48 kJ/kg body weight^0·75^ per h). The ATTD of organic matter, crude protein and crude fat was higher (*P* < 0·01) in the PF diet compared with the PFC diet, and lower (*P* < 0·01) for total carbohydrates. Exercise did not affect ATTD. Higher (*P* < 0·01) insulin-like growth factor 1 and leptin concentrations were measured when fed the PF diet compared with the PFC diet. Concentrations of insulin decreased (*P* < 0·01), whereas cortisol and ghrelin increased (*P* < 0·05), after exercise. There was no effect of diet on blood lactate and glucose, but higher (*P* < 0·001) lactate concentrations were measured in period 1 than in period 2. The results suggest that the o^13^CBT can be used in the field to estimate short-term EE in dogs during resting conditions. Higher ATTD and energy density of the PF diet may be beneficial when energy requirements are high.

Daily metabolisable energy (ME) requirements vary greatly among dogs depending on several factors, such as breed, body composition, age, physical activity and ambient temperature. For dogs living outdoors all year around, a decrease in ambient temperature of 20°C may increase the daily ME requirements by about 50 %^(^^1^^)^. Performance sled dogs, which also are working hard for many hours per d in cold environments, may at times have tremendously high energy requirements which can increase by up to eleven times their resting requirements^(^^2^^,^^3^^)^. Dogs performing endurance sports, such as sled dogs or hunting dogs, require energy-rich diets that are highly digestible, to provide a sufficient amount of energy in an amount of feed that the dogs are able to consume without having an inhibiting effect on performance, i.e. extra weight because of a filled stomach and intestines. As fat contributes more than twice the energy per g compared with protein and carbohydrates^(^^1^^)^, this macronutrient normally makes up the major part of the ME content (>50 %) in such diets. A recent study showed that dogs are dependent on carbohydrates to sustain prolonged periods of exercise^(^^4^^)^. The source of the carbohydrates being oxidised could not be identified in that study and could probably be derived from dietary protein. Other studies^(^^5^^–^^7^^)^ have shown beneficial effects on performance, e.g. increased stamina, of feeding carbohydrate-free diets, and there appears to be no need for carbohydrates in the diets as long as the diet contains enough protein. Nevertheless, the proportion of the macronutrients used as metabolic fuel during exercise is affected by the characteristics of the exercise, such as intensity and duration^(^^8^^)^, and therefore, it can be challenging to find a balance between macronutrients that results in optimal, efficient delivery of energy that matches the energy demands of the dog.

When evaluating energy and nutrient requirements, estimation of the energy expenditure (EE) of dogs in their daily life is important. Previous studies, in human subjects^(^^9^^)^, dogs^(^^10^^,^^11^^)^ and ponies^(^^12^^)^, have shown that the oral [^13^C]bicarbonate technique (o^13^CBT) can be used as a minimally invasive method to determine EE. The ‘gold standard’ method for measurement of EE is indirect calorimetry, i.e. measurement of O_2_ consumption (*R*O_2_) and CO_2_ production (*R*CO_2_). However, this method requires that the subject is confined to a respiration chamber or a special hood/mask. The o^13^CBT has been validated in dogs and it has been demonstrated that the method provides similar results to indirect calorimetry and that it can be used for the estimation of short-term EE of dogs in the fasted state and under resting conditions^(^^10^^)^. The objective of this study was to apply the o^13^CBT for determination of short-term EE in resting sled dogs under field conditions. The effects of diets with different fat:carbohydrate ratios on EE and plasma hormone concentrations were investigated under resting conditions. Furthermore, the effect of diet on the apparent total tract digestibility (ATTD) after days with rest or days with exercise was evaluated, as well as whether plasma hormone concentrations, blood lactate and glucose change with exercise.

## Materials and methods

The experimental procedures were in agreement with the Norwegian National Animal Research Authority, and were performed in accordance with institutional and national guidelines for the care and use of animals (Norwegian Animal Welfare Act, and the Norwegian Regulation on Animal Experimentation). The experiment was carried out at a sled dog kennel in Harestua, Oppland, Norway, as a cross-over study with two periods, each of 3 weeks. Each period included 14 d of adaptation and 1 week of sample collection. The experimental period started on 24 October. The outdoor temperature decreased over time, being around 4°C and with a relative humidity (rh) of approximately 70 % in the first period at the time of sample collection. In the second period, the outdoor temperature had decreased to about −16°C and the rh was approximately 45 %. Ambient temperature and rh were measured daily in the weeks with sample collection, between 10.00 hours until approximately 15.00 hours with a digital thermo-hygrometer (TFA Dostmann GmbH & Co. KG).

### Animals

A total of eight privately owned Alaskan Husky dogs, three males and three females, 15 months of age, and two adult males of 2 and 4 years, were included in the present study. Their mean start body weights (BW) were 19·5 (sd 1·6) kg, and all dogs remained healthy during the study. The dogs were kept outdoors at all times. Each dog had its own dog house of about 1 m^2^ and was confined to its respective kennel area. The floor was covered with a thick insulating layer of straw when the temperature decreased to around 0°C. Bowls with fresh water were attached to each dog house and the water was changed daily. The dog houses were placed in rows, about 5 m apart from each other. The area around the dog houses was cleaned for stools daily.

### Exercise

All dogs were simultaneously exercised in harness by pulling a four-wheeled vehicle put in neutral position. In general, the dogs were running with an average speed of approximately 16–18 km/h for a distance of 12 km on 4 d/week from 15 August, when the training season started (i.e. 10 weeks prior to the study). The distance at some training sessions could be longer, but in the study this was compensated by 1 d less with training, hence, the total distance per week was similar, i.e. 48 km. The positions in the group could vary slightly during each training session. In the sample collection weeks, the dogs were running 12 km/d on Monday, Tuesday, Thursday and Friday.

### Diets

Prior to the study, the dogs were fed approximately 150 g of a dry commercial food (Eukanuba^®^ Premium Performance, Mars Inc.; 30 % crude protein, 20 % crude fat) complemented with fatty by-products from pigs. The supplement of by-products was adjusted for each dog according to the trainer's evaluation of its body condition. Two wet diets were formulated to be used in this study and each diet was fed to four of the dogs at a time in the cross-over study. One diet was based on only protein- and fat-containing ingredients, resulting in only 1 % of ME being derived from carbohydrate (the PF diet). The other diet included nearly the same amount of protein, but the fat content was lower compared with the PF diet and instead 16 % of the ME was made up of carbohydrates in the form of precooked wheat (the PFC diet). Both diets were high-energy diets for physical performance and contained higher fat levels than those applied for sedentary dogs. The ingredients were raw by-products from salmon, poultry, pigs and cattle. The ingredient and chemical compositions of the diets are presented in Table 1. All ingredients were minced to 0·5 cm with a grinder and mixed in a paddle mixer. After the first mixing, a vitamin and mineral premix (5 g/kg feed) (Normin A/S) and yttrium oxide (Y_2_O_3_; 0·83 g/kg feed) for determination of ATTD were added in the paddle mixer and then the ingredients were mixed once more. The feed was packed in plastic bags in 1 kg portions, stored at – 20°C and thawed just before feeding. The dogs were fed 1·0–1·2 kg feed once per d, after finishing the exercise and sample collection. The amount of feed was estimated to cover the dogs’ daily requirements of ME under the prevailing conditions (about 1000 kJ/kg metabolic body size (BW^0·75^) per d) and was adjusted by the trainer subjectively. Due to the cold weather in period 2, the water source along the training route had frozen. Warm water (about 37°C) mixed with a small amount of the feed to a soup was therefore given to the dogs prior to the exercise. The dogs were allowed to drink from a bowl for 20–30 s, corresponding to about 0·2–0·3 litres.

**Table 1.** Ingredients, DM, chemical composition, metabolisable energy (ME) content and protein–fat–carbohydrate ratios of the experimental protein–fat–carbohydrate (PFC) and protein–fat (PF) diets

	Experimental diets	
	PFC	PF
Ingredient composition (g/kg diet)		
Salmon by-products	220	220
Chicken by-products	400	335
Beef/pig by-products		270
Cattle rumen	220	170
Precooked wheat	155	
Vitamin and mineral mixture*	5	5
Yttrium oxide	0·83	0·83

DM (g/kg diet)	454	410
Chemical composition (g/kg DM)		
Ash	75	81
Crude protein	339	369
Crude fat	323	514
Carbohydrates		
Starch	196	2
Dietary fibre	73	20
NSP	35	4
Lignin	38	16
Energy		
ME (MJ/kg DM)†	22·0	26·9
Protein:fat:carbohydrate ratio (% of ME)	26:58:16	24:75:1

* Containing per kg mixture: vitamin A (retinol), 1·05 mg; vitamin D_3_, 12·5 μg; vitamin E, 150·00 mg; thiamin, 1·50 mg; riboflavin, 2·80 mg, vitamin B_6_, 1·80 mg; vitamin B_12_, 0·02 mg; niacin, 9·00 mg; folic acid, 0·20 mg; pantothenic acid, 10·00 mg; biotin, 1500 mg; choline, 250·00 mg; vitamin C, 15·00 mg; Fe, 118 mg; Mn, 20 mg; Zn, 30 mg; Cu, 3·8 mg; I, 0·4 mg; Se, 0·16 mg.

† Determined by conversion factors (18·4 kJ/g protein, 39·8 kJ/g fat and 17·6 kJ/g carbohydrates) multiplied by the respective apparent total tract digestibility values for protein, fat and carbohydrates (see Table 2).

**Table 2.** Apparent total tract digestibility (%) of main nutrients measured in dogs* fed the experimental protein–fat–carbohydrate (PFC) and protein–fat (PF) diets, after 2 d with rest at the kennel and after 2 d with exercise† (Least square means (LS-means) and 95 % confidence intervals)

	Diet				Activity					
	PFC		PF		Rest		Exercise		*P*	
	LS-mean	95 % CI	LS-mean	95 % CI	LS-mean	95 % CI	LS-mean	95 % CI	Diet	Activity
Organic matter	89·9	88·7, 91·3	95·2	94·0, 96·5	92·6	90·8, 94·4	92·0	90·3, 93·8	<0·001	NS
Crude protein	91·3	90·2, 92·3	94·3	93·2, 95·3	92·8	91·8, 93·9	92·7	91·6, 93·7	<0·01	NS
Crude fat	98·6	98·3, 98·8	99·3	99·0, 99·5	98·7	98·6, 99·1	99·0	98·7, 99·2	<0·01	NS
Total carbohydrates	76·7	70·3, 82·5	49·6	43·2, 55·9	77·8	67·7, 88·0	74·9	64·7, 85·1	<0·001	NS

* Number of observations per treatment: *n* 8.

† Sled runs, 12 km/d with an approximate speed of 16–18 km/h.

### Data collection

Daily food intake and running distance were recorded throughout the experimental period. BW were measured at the start and the end of the experiment. Other data collection, i.e. blood samples, measurements of EE, heart rate (HR), rectal temperature (*T*_R_) and faeces collection, was performed in the third week of each period. The evening before faeces collection, which was done in the mornings after 2 d with rest and after 2 d with exercise, the areas around the dog houses were cleaned to ensure that the faeces collected were excreted after the latest meal.

During exercise, HR and blood samples were only taken from four dogs at a time, two dogs from each treatment group, to minimise the time for stopping.

### Measurement of short-term energy expenditure

The dogs were given an oral dose of ^13^C-labelled sodium bicarbonate (115 mg NaH^13^CO_3_, 98 atom% ^13^C; Sigma-Aldrich), mixed with a small amount of liver pâté (about 20 g). Expired air was then collected for ^13^C:^12^C isotope ratio analysis by using a mask (anaesthetic mask L; Jørgen Kruuse A/S) with a two-valve non-rebreathing system (Hans Rudolph, Inc.) and breath bags (Wagner Analysen Technik GmbH). Before the tracer administration, a baseline sample of expired air was taken from each dog, and, thereafter, samples were collected at 5, 10, 15, 20, 30, 40, 60, 90, 120 and 180 min after tracer administration. Each sampling lasted for 10–15 s.

### Heart rate recording

HR was recorded using Polar^®^ HR monitors (with R-R interval recording), consisting of electrode belt and transmitter W.I.N.D. and the HR monitor RS800 and RS800cx (Polar^®^Electro). The electrode belt was placed around the dogs’ chest and the fur was moistened with a transmission gel (Elektro-Stim) to ensure signal transmission to the electrodes. A custom-made protection belt was attached to the electrode belt. During days of rest, HR was recorded for approximately 40–50 min from four dogs at a time, during the same period of time as the measurements of EE, when the dogs were confined to their respective dog house. During days of exercise, HR recording started 10 min before the start and continued until 20 min after the training session ended.

### Rectal temperature

*T*_R_ was measured using a medical thermometer (Microlife AG), before and after the exercise sessions.

### Blood sampling

Blood was sampled for hormone analyses from the dogs in the post-absorptive state, before the measurements of EE, by cephalic vein puncture into heparinised tubes (5 ml Venoject^®^ tubes; Terumo Europe N.V.). Before taking the blood samples, a small area was shaved and cleaned with alcohol. Samples of blood for hormone analyses were also collected before the start of the exercise and after 12 km. Additionally, before the start, after 6 km and after 12 km, whole-blood glucose and lactate concentrations were measured by hand-held lactate (EKF Lactate Scout^+^ lactate analyser; EKF Diagnostics Holdings plc) and glucose (Accu-Check Aviva; Roche Diagnostics) analysers. The tubes with collected blood were centrifuged (Hettich centrifuge, EBA20) immediately after sampling at 2000 ***g*** for 15 min and the plasma was stored at −20°C until analysis for the plasma concentrations of insulin-like growth factor 1 (IGF-1), insulin, leptin, cortisol and total ghrelin.

### Analyses

In order to assess the ^13^C kinetics of CO_2_ in expired air, the collected breath samples were analysed for ^13^C:^12^C isotope ratios, as well as the CO_2_ concentration in the sample, by connecting the breath bags to an IR ^13^C isotope spectrometer (IRIS; Wagner Analysentechnik). The minimum level for reliable ^13^C analysis was a CO_2_ concentration of 0·3 vol%. Results of measured ^13^C:^12^C ratios of the samples were reported as relative difference from the international Pee Dee belemnite (PDB) reference standard, expressed by delta (*δ*) values in parts per million (ppm; ‰).

Feed and faeces were analysed according to the Association of Official Analytical Chemists (AOAC) (1990). DM was determined by drying to constant weight (24 h at 105°C) and samples were incinerated at 525°C for 6 h for determination of ash. Organic matter was calculated as: DM – ash. N was determined by the Kjeldahl technique (Tecator-Kjeltec system 1035; Tecator AB) and crude protein was calculated as: N × 6·25. Crude fat was determined after extraction with petroleum ether and acetone in an accelerated solvent extractor (ASE 200; Dionex). In the diets, starch was analysed by an enzymic–colorimetric method^(^^13^^)^ and dietary fibre was calculated as the sum of total NSP and Klason lignin^(^^13^^)^. Klason lignin was measured gravimetrically as the sulfuric acid insoluble residue^(^^14^^)^. Total carbohydrate content in faeces was calculated by difference: Total carbohydrate = DM − (crude protein + crude fat + ash). Yttrium in freeze-dried feed and faeces was solubilised according to the following procedure. Samples of 100 mg were combusted at 500–550°C for 4–5 h and subsequently mixed with 0·75 ml phosphoric acid (850 g/l) and 1 ml potassium bromate (45 g/l) and boiled for 10 min. The samples were cooled for 2–5 min at 20°C and then mixed with 5 ml of 5 g/l calcium chloride solution. Yttrium was analysed by inductivity coupled plasma mass spectrometry (ICP-AES analysis, Perkin Elmer Optia 3000 DV; Perkin Elmer) at 371 nm.

The hormone concentrations in plasma were analysed by RIA at the University of Western Australia, Perth. Plasma leptin was measured in duplicate using a double-antibody RIA method^(^^15^^)^. The measurements were made using human leptin; the first antibody was raised in a rabbit and the second in a donkey. All samples were processed in a single assay and the detection limit was 0·05 ng/ml. The assay included six replicates of three control samples containing 0·44, 0·81 and 1·7 ng/ml, which were used to estimate intra-assay CV of 5·6, 3·2 and 7·1 %, respectively. Plasma insulin was assayed in duplicate by a double antibody RIA^(^^16^^)^; the measurements were made using bovine insulin. The first antibody was raised in guinea-pigs and the second in goats. All samples were processed in a single assay and the detection limit was 2·51 µU/ml (17·43 pmol/l). In all, six replicates of three control samples containing 2·51, 5·24 and 10·89 µU/ml (17·43, 36·39 and 75·63 pmol/l) were included in the assay and were used to estimate intra-assay CV of 5·2, 3·6 and 6·0 %, respectively. Plasma IGF-1 was assayed in duplicate by a double-antibody RIA method with human recombinant IGF-1 (ARM4050; Amersham-Pharmacia Biotech) using rabbit antiserum and donkey antiserum following the protocol for acid-ethanol extraction and cryoprecipitation^(^^17^^)^ in plasma samples diluted by 1:40. The samples were processed in a single assay with a detection limit of 0·05 ng/ml. In all, six replicates of two control samples containing 0·16 and 1·16 ng/ml were included in the assay and were used to estimate intra-assay CV of 7·2 and 3·9 %, respectively. Plasma cortisol concentrations were measured in duplicate 50-μl aliquots after extraction with 2 ml of dichloromethane using an RIA based on separation with dextran-coated charcoal; the first antibody was raised in a rabbit^(^^18^^)^. All samples were processed in a single assay as duplicate 50-μl aliquots of plasma and the detection limit was 3·5 ng/ml. The assay included six replicates of two control samples containing 23·10 and 175·67 ng/ml, which were used to estimate intra-assay CV of 6·4 and 6·1 %, respectively. The concentration of total ghrelin was measured by the double-antibody RIA method of Miller *et al*.^(^^19^^)^. All samples were measured in a single assay in duplicate 50-μl aliquots of plasma; the detection limit was 0·40 pg/ml. The assay included six replicates of two control samples containing 115·6 and 1565 pg/ml, which were used to estimate intra-assay CV of 1·4 and 4·3 %, respectively. All assays were validated internally with serum dilutions for each assay.

### Calculations

The kinetics of the isotope ^13^C in breath CO_2_ was evaluated on the basis of the results from the ^13^C IR analysing system. Data were converted from *δ*^13^C into ^13^C abundance (*A*) (ppm), using the following equation:
1
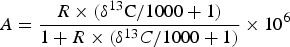

where *R* is the ratio of ^13^C to ^12^C of the international PDB standard (*R* 0·0112372), and *δ*^13^C is the relative ^13^C:^12^C ratio (‰) of the samples. The ^13^C enrichment (*E*_t_) (ppm) of expired CO_2_ was calculated as follows:
2
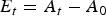

where *A*_*t*_ is the ^13^C abundance of the sample at the time *t* (min) after the tracer administration, and *A*_0_ is the basal ^13^C abundance before tracer administration. The *R*CO_2_ (mol/min) was calculated from the ^13^C data using the following equation^(^^20^^)^:
3
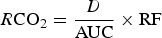

where *D* is the tracer dose administrated (mol), AUC is the area under the ^13^C enrichment-time curve (ppm × min), and RF is the fractional ^13^C recovery in breath CO_2_. Based on prior studies^(^^10^^,^^11^^,^^21^^)^, an estimate of 0·72 was used for the RF in this study. The AUC was determined by using the compartmental modelling module in the SAAM II computer software^(^^22^^)^. A compartmental model was created in order to represent the experimental conditions and to model the best-fit curve of the ^13^C enrichment-time data. The model consisted of three compartments illustrating the distribution and excretion of ^13^C after oral administration of NaH^13^CO_3_ as tracer. Compartment 1 represented the bicarbonate located in blood and well-perfused tissues which is turning over rapidly. The second compartment represented slow turning-over bicarbonate, i.e. bicarbonate of less well-perfused tissues and carbon in long-lived compounds such as skeletal muscle proteins. The third compartment represented the gastrointestinal tract which the [^13^C]bicarbonate crosses after oral administration of the tracer. The transfer and exchange of ^13^C (and ^12^C) between the different compartments were described in the model with rate constants (*k*, per min)^(^^12^^)^.

**Fig. 1.**
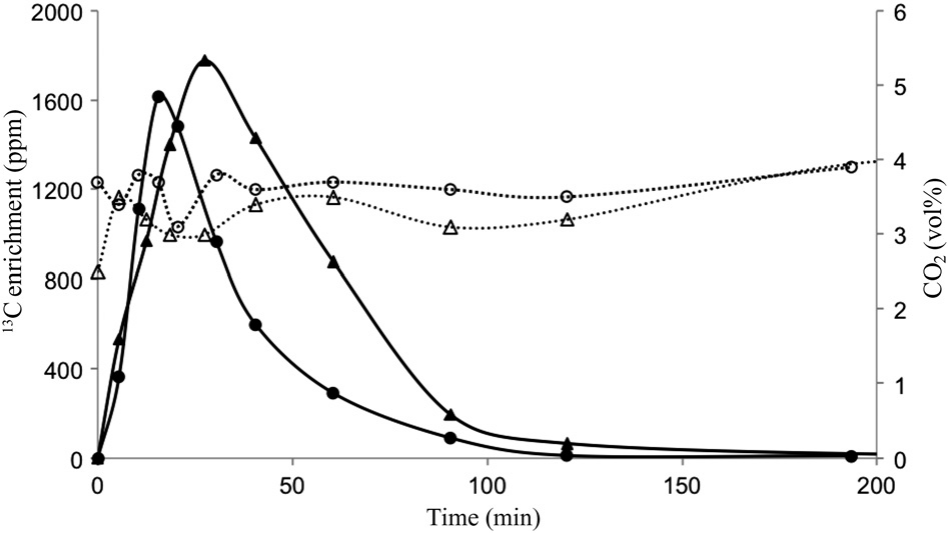
^13^C kinetics after administration of the ^13^C tracer to the same dog during resting conditions in period 1 (–▲–) and period 2 (–●–). Concentrations of carbon dioxide in the analysed breath bag during resting conditions in the same dog in period 1 (--△--) and period 2 (--○--). ppm, Parts per million.

Measurements lasted for approximately 2–3 h, i.e. the time for the ^13^C enrichment-time curve to return to baseline level (Fig. 1), and the resulting CO_2_ production was standardised to litres/h. As only the production of CO_2_ and not the O_2_ consumption is assessed with the o^13^CBT, an estimate of the respiratory quotient (RQ) is needed for the estimation of EE. Based on previous results in fasted dogs^(^^10^^,^^11^^)^, an estimate of 0·77 was used for the RQ and EE (kJ/h) was estimated according to the equation^(^^23^^)^:
4
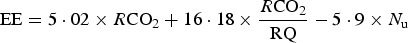

The contribution of the urinary N excretion (*N*_u_) to the value of EE is usually smaller than 1 %^(^^24^^)^. Therefore, urine was not collected and the last term of equation (4) was neglected. The results were finally expressed as kJ/kg BW^0·75^ per h.

The ATTD values of main nutrients were calculated based on the analysed concentration of nutrients (Ntr) and yttrium (*Y*) in diet and faeces, using the following formula:
5
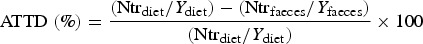

The dietary ME was determined by conversion factors, 18·4 kJ/g protein, 39·8 kJ/g fat and 17·6 kJ/g carbohydrates^(^^25^^)^ multiplied with the respective ATTD values for protein, fat and carbohydrates.

### Statistical analyses

Data were statistically analysed using the MIXED procedure in SAS^®^ (version 9.3; SAS Institute Inc.). Measured parameters were tested for difference using diet, period and their interactions as fixed effects, and dog as random effect. For hormonal effects, exercise (before and after exercise) and for the glucose and lactate measurements, distance (0, 6 and 12 km), were also included as fixed effects. The ATTD values were tested for difference using diet, activity (rest and exercise), period and their interactions as fixed effects and dog as random effect. The models were then reduced for non-significant interaction effects. Results are presented as least square means (LS-means) with their 95 % CI. Effects were considered significant if *P* < 0·05.

## Results

### Feed intake and body weights

The PF diet contained more ME per kg DM than the PFC diet (26·9 and 22·0 MJ/kg DM in the PF and the PFC diets, respectively). The DM content was higher in the PFC diet (45·1 %) than in the PF diet (41·0 %). The intake of ME when the dogs were fed the PF diet was 1261 (95 % CI 1171, 1351) and 1153 (95 % CI 1075, 1231) kJ/kg BW^0·75^ per d when they were fed the PFC diet. The mean BW at the end of the experiment was 21·9 (sd 2·2) kg, meaning that the dogs had gained on average 2·4 kg BW during the experimental periods. Therefore, the calculated ME intake per BW^0·75^ in period 1 was based on their start BW and in period 2 on their BW registered at the end. The daily ME intake was higher (*P* *<* 0·05) in the first period than in the second, being 1301 (95 % CI 1211, 1392) and 1113 (95 % CI 1035, 1991) kJ/kg BW^0·75^ per d in periods 1 and 2, respectively.

### Apparent total tract digestibility

The ATTD of organic matter, crude protein, crude fat and total carbohydrates, the effect of dietary treatment and activity are reported in Table 2. There was an effect (*P* < 0·01) of diet on the ATTD values for organic matter, crude protein and crude fat, which all were higher for the PF diet than the PFC diet. The ATTD value for total carbohydrates was higher (*P* < 0·01) for the PFC diet, compared with the PF diet. Exercise did not affect the ATTD for organic matter or any of the main nutrients analysed. Only the ATTD of crude protein differed between the two periods, and was higher (*P* = 0·04) in period 2 (93·9 %) than in period 1 (91·2 %).

### Heart rate

There was no effect of diet on the average HR measured during resting conditions, but the recorded values were higher (*P* < 0·01) in period 2 than in period 1 (Table 3). HR measured during and after exercise was not affected by dietary treatment or period. The average HR increased from 161 (sd 19) beats per min (bpm) measured 10 min before the start to 287 (sd 16) bpm during exercise. At 20 min after exercise, the average HR had decreased to 177 (sd 9) bpm, i.e. they were still considerably higher than during rest.

**Table 3.** Heart rate, estimated carbon dioxide production and energy expenditure in dogs* fed the experimental protein–fat–carbohydrate (PFC) and protein–fat (PF) diets during days with rest at the kennel (Least square means (LS-means) and 95 % confidence intervals)

	Diet				Period					
	PFC		PF		1		2		*P*	
	LS-mean	95 % CI	LS-mean	95 % CI	LS-mean	95 % CI	LS-mean	95 % CI	Diet	Period
Heart rate (bpm)	123	115, 130	119	111, 128	113	104, 121	129	121, 138	NS	<0·01
CO_2_ production(litres/kg BW^0·75^ per h)	2·0	1·4, 2·6	2·4	1·8, 3·0	1·8	1·2, 2·4	2·6	2·0, 3·2	NS	<0·01
Energy expenditure (kJ/kg BW^0·75^ per h)	53	37, 69	63	47, 79	48	32, 64	68	52, 84	NS	<0·01

bpm, Beats per min; BW^0·75^, metabolic body weight.

* Number of observations per treatment: *n* 8.

### Rectal temperature

Diet did not affect the *T*_R_ of the dogs. Mean *T*_R_ measured before exercise in period 2 was higher (*P* = 0·001) than in period 1, with *T*_R_ of 38·5 (sd 0·4)°C (period 1) and 38·9 (sd 0·4)°C (period 2), respectively. *T*_R_ increased (*P* < 0·001) in all dogs as a response to the exercise, but more (*P* < 0·02) in period 1 than in period 2. *T*_R_ post-exercise was 40·1 (sd 0·5) and 39·7 (sd 0·6)°C in periods 1 and 2, respectively.

### Carbon dioxide production and energy expenditure

Results of the estimated *R*CO_2_ and EE for the two treatment groups in both periods are given in Table 3. The experimental diets did not affect the estimated *R*CO_2_ or EE, but the estimated values were higher (*P* < 0·01) in period 2 than in period 1.

### Blood samples

#### Plasma hormone concentrations

There was no effect of diet on insulin, cortisol or ghrelin concentrations (Tables 4 and 5). Dogs fed the PF diet had higher (*P* < 0·01) concentrations of IGF-1 and leptin than dogs fed the PFC diet. The measured plasma concentrations of insulin decreased (*P* < 0·01) with exercise, whereas cortisol and total ghrelin increased (*P* < 0·05) (Table 5). There was no effect of period on the concentrations of the measured hormones except for insulin, which was higher (*P* < 0·01) during resting conditions in the second than in the first period (Table 4).

**Table 4.** Plasma concentrations of insulin, insulin-like growth factor 1 (IGF-1), cortisol, leptin and total ghrelin measured in dogs* fed the experimental protein–fat–carbohydrate (PFC) and protein–fat (PF) diets during days with rest at the kennel (Least square means (LS-means) and 95 % confidence intervals)

	Diet				Period					
	PFC		PF		1		2		*P*	
	LS-mean	95 % CI	LS-mean	95 % CI	LS-mean	95 % CI	LS-mean	95 % CI	Diet	Period
Insulin (μU/ml)†	7·2	5·4, 9·0	6·3	4·5, 8·2	5·1	3·3, 6·9	8·4	6·6, 10·2	NS	<0·01
IGF-1 (ng/ml)	7·2	5·9, 8·5	10·4	9·1, 11·7	8·5	7·2, 9·8	9·1	7·8, 10·3	<0·01	NS
Cortisol (nmol/l)	91·9	68·2, 115·6	82·6	58·9, 106·4	80·1	56·3, 103·8	94·5	70·8, 118·2	NS	NS
Leptin (ng/ml)	0·36	0·30, 0·42	0·52	0·46, 0·59	0·43	0·37, 0·49	0·45	0·39, 0·52	<0·01	NS
Total ghrelin (pg/ml)	1212	962, 1463	1039	789, 1290	1134	884, 1384	1118	867, 1368	NS	NS

* Number of observations per treatment: *n* 8.

† To convert to pmol/l, multiply by 6·945.

**Table 5.** Plasma concentrations of insulin, insulin-like growth factor 1 (IGF-1), cortisol, leptin and total ghrelin in dogs* fed the experimental protein–fat–carbohydrate (PFC) and protein–fat (PF) diets, measured before and after exercise† (Least square means (LS-means) and 95 % confidence intervals)

	Diet				Exercise					
	PFC		PF		Before		After		*P*	
	LS-mean	95 % CI	LS-mean	95 % CI	LS-mean	95 % CI	LS-mean	95 % CI	Diet	Exercise
Insulin (μU/ml)‡	9·1	6·8, 11·4	7·1	4·8, 9·4	10·1	7·8, 12·4	6·1	3·8, 8·4	NS	<0·01
IGF-1 (ng/ml)	6·9	6·2, 7·6	9·5	8·8, 10·2	8·2	7·5, 8·9	8·2	7·5, 8·9	<0·001	NS
Cortisol (nmol/l)	139	113, 166	146	120, 173	116	90, 142	170	144, 196	NS	<0·01
Leptin (ng/ml)	0·35	0·32, 0·39	0·48	0·44, 0·51	0·42	0·38, 0·44	0·41	0·39, 0·45	<0·001	NS
Total ghrelin (pg/ml)	1404	1033, 1776	1298	927, 1670	1225	853, 1596	1478	1107, 1850	NS	0·02

* Number of observations per treatment: *n* 8.

† Sled runs, 12 km/d with an approximate speed of 16–18 km/h.

‡ To convert to pmol/l, multiply by 6·945.

#### Blood glucose and lactate during exercise

**Fig. 2.**
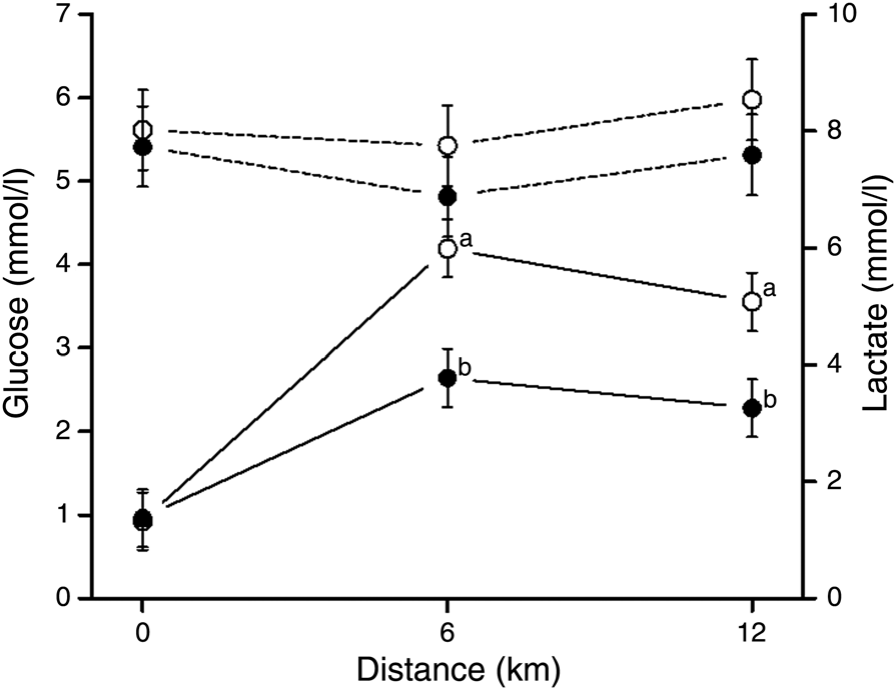
Blood glucose (---) and lactate (––) concentrations measured in dogs (*n* 8) during exercise in period 1 (○) and period 2 (●). Blood samples were taken pre- (0 km), midway (6 km) and post-exercise (12 km). Values are means, with standard errors represented by vertical bars. ^a,b^ Mean values with unlike letters were significantly different between the two periods (*P* < 0·05).

There were no effects of diet or period on blood glucose concentrations and measured levels were rather stable during exercise (Fig. 2). Blood lactate increased with exercise in both periods, but higher (*P* < 0·01) concentrations were measured in the first period after running 6 and 12 km compared with in period 2 (Fig. 2). The measured lactate concentrations were increased (*P* < 0·001) after 6 km, but remained stable to the last measurement at 12 km.

## Discussion

In the present study, the o^13^CBT was used on dogs under field conditions. The estimated EE from measurements under resting conditions at the kennel varied greatly between the dogs. However, individual variation was expected. Dogs were of similar genotype and measured under the same conditions, but other factors such as body composition^(^^26^^,^^27^^)^ and activity while kennelled probably differed and influenced the results. EE was not affected by the dietary treatment. However, EE was higher in all dogs in period 2 (68 kJ/kg BW^0·75^ per h) compared with period 1 (48 kJ/kg BW^0·75^ per h). One explanation for this increase could probably be the changed weather conditions. The ambient temperature during the measurements was around 4°C in the first period, and decreased to around −16°C and with snow in period 2. The increase in EE and HR in the second period indicates that the ambient temperature was below the lower critical temperature of the dogs, i.e. the ambient temperature below which metabolic rate increases to maintain body temperature, as this would lead to increased metabolic heat production to maintain homeothermy^(^^1^^,^^3^^,^^28^^)^. Improved fitness may be another explanation. The concentrations of blood lactate were not affected by the two diets, but increased for all dogs during exercise. The degree of increase though was higher in the first period compared with the second. As a metabolite deriving from anaerobic pathways, the concentration of lactate serves as an indicator of the fitness and level of the exercise and the oxidative capacity^(^^29^^)^. The lower blood lactate concentrations measured in period 2 could indicate that after three more weeks of exercise, the dogs had become better conditioned and, consequently, that additional energy was spent to develop and maintain the muscle proteins necessary to convey greater aerobic capacity of conditioned muscles. Substantially lower lactate concentrations than in this study were measured in a previous study^(^^4^^)^ and in which the concentrations were not different between exercise and resting conditions. However, the dogs in that study were older (5 ± 1 years) than in the present study (six out of eight dogs were 15 months old) and they were certainly in better physical condition due to a longer period of training prior to the experiments.

EE in sedentary dogs and where the ambient temperature was about −10 to −30°C has been measured to be 46 kJ/kg BW^0·75^ per h^(^^3^^)^ and 35 kJ/kg BW^0·75^ per h^(^^30^^)^, when measured by the doubly labelled water (DLW) method. The DLW method measures EE over a longer period of time, i.e. several days, meaning that the estimated EE value is an average of all activities during the measurement period. Contrary to the DLW method, the o^13^CBT is based on short-term measurements of 2–3 h. This is an advantage when measuring the EE in order to get more knowledge of the energy costs in specific situations. The estimated EE values in this study were higher than found previously. This can be explained by the measurements being performed during the time of the day when the dogs most probably were the most active. However, all dogs gained BW during the experimental period. This indicates that the ME intake of 1301 and 1113 kJ/kg BW^0·75^ per d in periods 1 and 2, respectively, was higher than the daily EE of the dogs. As they were only weighed at the start and at the end of the experiment, it cannot be elucidated whether the dogs gained BW continuously over both periods or only in period 1, where the ME intake and the ambient temperature were higher than in period 2.

In the present study, an estimate of 0·72 was used for the RF and 0·77 for the RQ. These estimates are based on dogs, also fasted over the night, but lying down resting throughout the measurements in a respiration chamber^(^^10^^,^^11^^)^. Diet and activity are factors that influence both the RF^(^^4^^,^^31^^,^^32^^)^ and RQ^(^^20^^)^ and for the accuracy of results, these estimates need to be further validated for the use of the o^13^CBT during different measurement conditions. An RQ of 0·71 reflects fat oxidation and an RQ of 1·0 oxidation of carbohydrates^(^^25^^)^. Thus, it could be expected that the RQ would differ between the dietary treatment groups when the dogs were in the fed state, because of higher fat oxidation for dogs fed the PF diet than when fed the PFC diet. If using the food quotient for the diets as an estimate for the RQ, as has been suggested previously^(^^33^^)^, the estimates would be 0·74 for the PF diet and 0·79 for the PFC diet. However, the dogs in this study were in the fasted state, which also induces higher fat oxidation than carbohydrate oxidation. Therefore the value of 0·77 was considered to be an appropriate estimate for the RQ in this study, as this RQ value was previously measured by indirect calorimetry in fasted dogs that were completely relaxed and also in dogs that were slightly more active during the measurements^(^^10^^,^^11^^)^. An error of ±0·1 of the estimate for RQ, however, i.e. if using estimates of 0·67 or 0·87, would lead to 9 % error in the estimation of EE^(^^12^^,^^34^^)^.

It has previously been demonstrated that Y_2_O_3_ can be used as an inert marker for determining ATTD in dogs^(^^35^^)^. The ATTD for organic matter, crude protein and crude fat were high for both diets. These high values reflected the high quality of the ingredients. However, higher values were measured in the PF diet compared with the PFC diet, which might be preferable for exercising dogs during the winter season when energy needs are markedly increased due to low ambient temperatures. The carbohydrates in the PFC diet consisted of 73 % starch and 27 % dietary fibres. The ATTD of starch is close to 100 %^(^^35^^)^ but the fermentability of dietary fibre from wheat is poor^(^^36^^)^, resulting in a lower ATTD of carbohydrates than crude protein and crude fat. Calculating the ATTD of total carbohydrates by difference may lead to inaccurate estimates in diets with low carbohydrate content like in the PF diet, as all the analytical errors will add up on this fraction^(^^37^^)^. The lower ATTD of organic matter in the PFC diet than the PF diet was a result of the differences in contents and ATTD of carbohydrates in the diets.

Exercise has been associated with changes in intestinal functions in dogs^(^^38^^,^^39^^)^ and it has been discussed whether ATTD is decreased by exercise^(^^40^^)^. If the ATTD is decreased, the ME of the diet would consequently be lower. However, there was no effect of exercise on ATTD of organic matter and the main nutrients in this study. Similar results were found previously in hunting dogs exercised at a moderate or light level^(^^40^^)^, according to the exercise severity scale suggested by Burger^(^^41^^)^ where low corresponds to exercise below 1 h/d and moderate exercise corresponds to 1–3 h/d. Although the type of exercise differed between these two studies in terms of intensity and duration, the exercise in the present study would also be classified as low to moderate according to the scale of Burger^(^^41^^)^. This suggests that light to moderate exercise does not affect the ATTD of the nutrients in dogs. However, more strenuous exercise for a longer period may affect the ATTD differently. As well, a higher feeding level, like for ultramarathon huskies, could possibly decrease the ATTD compared with the feeding level in this study.

The glucose concentrations measured in whole blood were rather constant and not affected by the two dietary treatments, exercise or by the two periods of measurement.

Higher concentrations of IGF-1 and leptin were observed in dogs fed the PF diet. Leptin is central in the regulation of appetite and energy metabolism and increased concentrations of this hormone increase the feeling of satiety^(^^42^^,^^43^^)^. The increase in cortisol and decrease in insulin concentrations are characteristic as a response to exercise, stimulating hepatic gluconeogenesis^(^^44^^,^^45^^)^. Higher cortisol concentrations were observed before the exercise compared with the samples taken during days with rest. This increase is likely an effect of excitement and has been observed previously in sled dogs before exercise^(^^46^^)^. Ghrelin stimulates the appetite and has an important role in energy homeostasis^(^^47^^)^. No difference in total ghrelin concentrations between the diets was observed, but the levels increased after exercise. The ghrelin concentrations in humans change during the day where it peaks before meal time and thereafter falls in proportion to the amount of energy ingested^(^^47^^)^. The same pre- and postprandial pattern of ghrelin has been found in dogs^(^^48^^)^. Hence, the increased levels observed after exercise in this study were probably associated with the anticipation of feed because the dogs were fed once daily, after the exercise. Whether the exercise affected the ghrelin concentrations could not be determined but similar concentrations in periods 1 and 2 indicate that exercise had no long-term effect on ghrelin. However, the concentrations of total ghrelin were measured in this study, hence, it could not be distinguished between acylated or unacylated ghrelin, which are involved in different physiological processes^(^^47^^)^, and it could not be determined if the proportion of acylated and unacylated ghrelin changed as a result of exercise.

### 

#### Conclusions

This study suggests that the o^13^CBT can be used in the field for estimation of short-term EE in dogs during resting conditions to get more knowledge of the energy costs in specific situations. The physiological changes recorded in this study were more likely to be associated with the change in ambient temperature and/or a longer period with regular exercise, than an effect of the two dietary treatments. The difference in fat:carbohydrate ratios between the diets was not sufficient to demonstrate any positive or negative effects when fed to dogs under the conditions prevailing in this study. The ATTD and energy density were higher in the PF diet than in the PFC diet, which may be beneficial when the energy requirements are high.
